# Identification and characterisation of the BPI/LBP/PLUNC-like gene repertoire in chickens reveals the absence of a LBP gene^☆^

**DOI:** 10.1016/j.dci.2010.09.013

**Published:** 2011-03

**Authors:** Shih-Chieh Chiang, Edwin J.A. Veldhuizen, Frances A. Barnes, C. Jeremy Craven, Henk P. Haagsman, Colin D. Bingle

**Affiliations:** a Academic Unit of Respiratory Medicine, Department of Infection and Immunity, University of Sheffield, Sheffield S10 2JF, UK; b Department of Infectious Diseases and Immunology, Faculty of Veterinary Medicine, Utrecht University, P.O. Box 80.165, 3508 TD Utrecht, The Netherlands; c Krebs Institute for Biomolecular Research, Department of Molecular Biology and Biotechnology, University of Sheffield, Sheffield S10 2TN, UK

**Keywords:** Innate immunity, BPI, LBP, PLUNC, Comparative genomics, Avian

## Abstract

Palate, lung and nasal epithelial clone (PLUNC) proteins are structural homologues to the innate defence molecules LPS-binding protein (LBP) and bactericidal/permeability-increasing protein (BPI). PLUNCs make up the largest portion of the wider BPI/LBP/PLUNC-like protein family and are amongst the most rapidly evolving mammalian genes. In this study we systematically identified and characterised BPI/LBP/PLUNC-like protein-encoding genes in the chicken genome. We identified eleven complete genes (and a pseudogene). Five of them are clustered on a >50 kb locus on chromosome 20, immediately adjacent to *BPI*. In addition to BPI, we have identified presumptive orthologues LPLUNCs 2, 3, 4 and 6, and BPIL-2. We find no evidence for the existence of single domain containing proteins in birds. Strikingly our analysis also suggests that there is no LBP orthologue in chicken. This observation may in part account for the relative resistance to LPS toxicity observed in birds. Our results indicate significant differences between the avian and mammalian repertoires of BPI/LBP/PLUNC-like genes at the genomic and transcriptional levels and provide a framework for further functional analyses of this gene family in chickens.

## 1 Introduction

LPS, a major component of the outer membrane of Gram-negative bacteria, is a potent immunogenic molecule to mammals. Exaggerated host immune response to LPS can lead to shock, multi-organ failure and death. LPS-binding protein (LBP) and bactericidal/permeability-increasing protein (BPI) are two extensively investigated proteins in mammals due to their central role in regulating LPS presentation to neutrophils and subsequent inflammatory response. Although structurally similar, LBP and BPI have opposing effects on LPS-mediated inflammatory response. LBP promotes inflammatory response by catalysing binding of LPS monomers to CD14 on the cell surface, and together with Toll-like receptor 4 (TLR-4), mediates activation of these cells and clearance of LPS from the host (Fenton and Golenbock, 1998; Hailman et al., 1994; Weiss, 2003). On the other hand, BPI is a bactericidal protein that also exhibits anti-inflammatory activity through high affinity binding with, and neutralisation of LPS, and which in conjunction with other host factors results in arrest of bacterial growth and phagocytosis by neutrophils (Weiss, 2003; Elsbach and Weis, 1998). In addition to LBP and BPI, this family of lipid transfer proteins also includes phospholipid transfer protein (PLTP) and cholesterol ester transfer protein (CETP), two proteins important in promoting the exchange of neutral lipids and phospholipids between the plasma lipoproteins (Masson et al., 2009). In keeping with their shared structure, both BPI and CETP have been shown to co-crystalize with lipids. BPI and CETP adopt two structurally homologous domains and each contains a hydrophobic pocket, which binds lipids (Beamer et al., 1997; Qiu et al., 2007). In keeping with the proposed lipid binding/transfer function of members of the BPI/LBP/CETP/PLTP family, insect juvenile hormone binding protein binds to the highly hydrophobic juvenile hormone with high affinity (Kolodziejczyk et al., 2008). Recently the crystal structure of *Epihyas postvittana* Takeout1 was solved with a bacterial lipid present in the conserved hydrophobic cleft (Hamiaux et al., 2009) further supporting a lipid binding role for proteins with this fold. Additional structurally related molecules have also been identified in the pacific oyster (Gonzalez et al., 2007), trypanosomes (Barker et al., 2008) and in worms (Oishi et al., 2009) confirming the widespread distribution of this fold throughout the animal kingdom.

Our identification and description of the palate, lung and nasal epithelial clone (PLUNC) proteins has greatly enlarged the BPI/LBP/CETP/PLTP protein family (Bingle and Craven, 2002, 2004; Bingle et al., 2004). In fact the PLUNC branch makes up the largest portion of the family which can therefore be called the BPI/LBP/PLUNC-like family. PLUNC was first described in the nasal epithelium of the mouse embryo and the trachea/bronchi of adult mice (Weston et al., 1999). We made the key observation that *PLUNC* belongs to a family of genes located in a single locus on human chromosome 20q11 (Bingle and Craven, 2002, 2004; Bingle et al., 2004; Wheeler et al., 2007). We showed that PLUNCs make up the largest branch of this family and can be classified into long PLUNC (LPLUNC) or short PLUNC (SPLUNC). LPLUNCs exhibit homology to both the N-terminal and C-terminal domains of BPI whereas SPLUNCs exhibit structural homology to the N-terminal domain of BPI (containing the LPS-binding motif) (Bingle and Craven, 2002, 2004; Bingle et al., 2004). We have previously shown that SPLUNCs evolved by partial duplication of a LPLUNC gene and have rapidly evolved to yield a diverse gene set in different mammals (Bingle and Craven, 2002, 2004; Bingle et al., 2004; Wheeler et al., 2007). Although the function of PLUNC proteins is not known, there has been accumulating evidence suggesting that they may play a role in host innate immunity. The findings that human SPLUNC1 binds to *Escherichia coli* LPS (Ghafouri et al., 2003) and that rat parotid secretory protein (the orthologue of human SPLUNC2) can complex with bacterial membranes (Robinson et al., 1997) support a role for direct interaction with LPS. There is conflicting data on a direct anti-microbial role for SPLUNCs but there is some evidence that SPLUNCs attenuate the growth of a range of organisms (Geetha et al., 2003; Chu et al., 2007; Haigh et al., 2008). The observation that PLUNCs are amongst the most rapidly evolving mammalian genes and the presence of species-specific members of the PLUNC family suggests evolution may have been driven through interaction with species-specific pathogens (Bingle et al., 2004; Wheeler et al., 2007).

On the basis of genomic and EST data available at the time we initially proposed that PLUNCs were restricted to mammals (Bingle et al., 2004). However, studies have characterised two PLUNC-related genes in chicken, Transiently Expressed in Neural Precursors (TENP) and Ovocalyxin-36. TENP is expressed in embryonic chicken brain and retina, specifically in postmitotic cells before they enter the stage of differentiation (Yan and Wang, 1998). The discovery of TENP predated our identification of PLUNCs but our phylogenetic analysis could not robustly place it within the PLUNC sub-branch of the wider family (Bingle et al., 2004). Proteomic analysis of hen egg white subsequently revealed the presence of TENP in a significant quantity (approximately 0.1–0.5%) (Guérin-Dubiard et al., 2006). Ovocalyxin-36 is another protein with sequence homology to LBP, BPI and PLUNC (Gautron et al., 2007). Ovocalyxin-36 was suggested to be an egg-shell specific protein and was shown to contain both the N-terminal and C-terminal BPI-like homology domains. The exon/intron structure of *Ovocalyxin-36* was also shown to be very similar to *LBP* and *BPI* (and consequently to PLUNCs). In addition, *Ovocalyxin-36* is located on chromosome 20, which is syntenic to the portion of human chromosome 20 (International Chicken Genome Sequencing Consortium, 2004) where *LBP* and *BPI* are located, suggesting a common ancestral origin before the divergence of mammalian and avian lineages (International Chicken Genome Sequencing Consortium, 2004). Proteomic analysis has detected Ovocalyxin-36 in the vitalline membrane, egg shell, uterus and red isthmus of the female reproductive tract (Mann, 2008). In addition to these two proteins chicken CETP and PLTP have also been cloned (Sato et al., 2007; Saarelaa et al., 2009).

On the basis of these observations and using the availability of an improved chicken genome assembly and the large chicken EST database we now report the systematic identification and expression analysis of BPI/LBP/PLUNC-like genes in the chicken. Our results indicate significant differences between the avian and mammalian repertoires of BPI/LBP/PLUNC-like genes at the genomic and transcriptional levels. Key amongst these are the apparent lack of an avian LBP gene and the lack of any single BPI-domain containing (SPLUNC-like) proteins. These observations provide a framework for further functional analyses of this gene family in chickens.

## 2 Materials and methods

### 2.1 Database searching

The chicken Ensembl database (release 49, March 2008) was initially searched for known and predicted proteins containing the InterPro LBP_BPI_CETP protein domain (IPR001124). We also searched for Genscan gene predictions and performed an extensive sequence based iterative analysis using NCBI BLAST as previously outlined (Bingle et al., 2004).

To identify the genomic localisation of each gene we utilised data from Ensembl as well as direct mapping of the predicted transcripts onto the genomic sequence using the BLAST-like alignment tool (BLAT) (Kent, 2002). Gaps in the current chicken genome assembly (v.2.1 May 2006) that affected the prediction of exon numbers and sizes for a number of the genes, were closed by PCR based amplification, cloning and sequencing as outlined below. For phylogenetic analysis and the assignment of orthology we also collected a range of related sequences from a range of sources.

### 2.2 EST and genomic sequencing, PCR primer design and genomic PCR

We aimed to generate confirmatory evidence for the existence of all of the identified genes. Where ESTs partially supporting these genes existed in the database (Table 1) these were obtained and fully sequenced using an ABI DNA Analyser 3730 capillary sequencer. The sequences of the full-length ESTs and predicted transcripts were used as templates for designing internal oligonucleotide primers for use in PCR-based cloning so as to obtain the complete transcript and genomic sequences of each predicted gene. We used Primer3 (http://primer3.sourceforge.net/) to select primer sets for each transcript (Table 2). All primers were chosen with a default annealing temperature of 60 °C. Where gaps were noted in the genomic assembly we generated overlapping primer sets and used these for PCR with high fidelity Taq polymerase (AccuPrime, Invitrogen) using chicken genomic DNA as a template. The resultant products were cloned in pCRII Topo (Invitrogen), mini prepped and sequenced as above. Large inserts were sequenced with internal specific primers as required. The primers used for this amplification and for sequencing are also listed in Table 2.

### 2.3 Sequence analysis

Using the fully sequenced genomic and transcript information, the exon numbers and sizes of each PLUNC gene were determined. The predicted protein sequences were generated from sequences of full-length ESTs wherever possible, or from a combination of EST sequences, Ensembl predicted transcript sequences and NCBI GenBank predicted peptide sequences. Six-frame translations were generated by ExPASy Translate Tool and searched against the NCBI peptide sequences using protein BLAST (Altschul et al., 1990) to identify the best prediction. This analysis allowed the resolution of all intron exon boundaries.

The full-length protein sequences were then ClustalW aligned (Thompson et al., 1994) with sequences of human and mouse LPLUNCs and with a selection of BPI/PLUNC related proteins from cow, frog, some fish species and a single oyster protein. The sequences used in this analysis and their origins are shown in Fig. 2. A neighbour-joining phylogenetic tree was generated to assess the general branching of the BPI/PLUNC family and to identify the orthologues of the chicken proteins. The underlying tree was created using clustalW, a preliminary representation was made using phylip (Felsenstein, 1989) and the final tree was created using in-house software.

### 2.4 Expression analysis

We established RT-PCR conditions for each chicken transcript using cDNA generated from pooled commercial RNA samples (Zyagen, USA). Tissues used were brain, heart, lung, kidney, liver, small intestine, colon, testes and skeletal muscle. Reverse transcription was performed using standard protocols, in a total volume of 20 μl using an oligo-dT primer and 1 μg of pooled total RNA. PCR reactions were performed using 1 μl of each reaction product. PCR conditions and the required number of cycles were determined empirically. Reaction products were resolved on 1–2% TAE agarose gels and subsequently cloned and sequenced for verification purposes.

To investigate expression of each gene in a wider range of tissues total RNA was extracted from 40 tissues of healthy ROSS 308 broiler chicken using TRIzol (Invitrogen) and Magnalyser Green Beads (Roche Diagnostics) following manufacturer's directions. 1 μg of RNA was DNAse I-treated (Fermentas) and was reverse transcribed using iScript cDNA synthesis kit (Bio-Rad Laboratories). The cDNA was standardised using primers for the housekeeping gene β-actin in PCR, and all subsequent PCRs were done using FastStart DNA Taq polymerase, dNTP pack (Roche Diagnostics). Thermocycling comprised of initial denaturation of 95 °C for 5 min, followed by 40 cycles of 95 °C for 40 s, 60 °C for 1 min, 72 °C for 1 min, then a final extension of 72 °C for 5 min. The outcome was assessed by 1% TAE agarose electrophoresis using ethidium bromide for visualisation under an ultraviolet transilluminator.

## 3 Results

### 3.1 Identification of chicken BPI/LBP/PLUNC-like family members

As comparative genomic analysis of the mammalian BPI/LBP/PLUNC-like genes has shown low sequence identities between orthologous pairs (Bingle et al., 2004), identification of PLUNC orthologues in more divergent vertebrate species including the chicken relies primarily upon conservation of functional protein domains (the LBP_BPI_CETP domain) and exon sizes.

Searching of the Ensembl database for known and predicted proteins containing the InterPro LBP_BPI_CETP protein domain (IPR001124) identified eleven potential genes. Noting that eight out of eleven of the predicted genes cluster on chromosome 20, which is partially syntenic to chromosome 20 in human and chromosome 2 in mouse, where *BPI* and *LBP* are located, we searched for additional candidate genes in the Genscan gene predictions database. This search identified one additional gene prediction (which also has the Ensembl identity, ENSGALG00000006693). To complete the bioinformatic analysis we performed an extensive sequence based iterative analysis using NCBI BLAST.

**Fig. 1:**
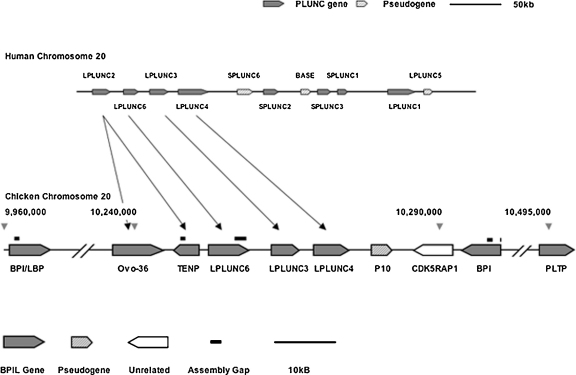
Organisation of the BPI/LBP/PLUNC-like gene locus in chickens. Schematic representation of the human PLUNC and chicken BPI/LBP/PLUNC-like loci. The lower panel indicates the organisation of the chicken locus on Chromosome 20, whereas the upper panel represents the human locus (adapted from Wheeler et al., 2007). Arrows between the two loci indicate orthologous relationships described in the text. The position of the locus on the chromosome is indicated by the grey arrow-heads and nucleotide numbers of the assembled chicken chromosome, obtained from the UCSC BLAT genome browser (http://genome.ucsc.edu/). The solid bars above the locus indicate the positions of gaps in the assembly that were closed in this study. The cross hatches at either end of the locus indicate breaks in the sequence introduced for clarity. The arrowed end of each gene indicates the direction of the ORF. Intact BPI/LBP/PLUNC-like (BPIL) genes are shaded while the pseudogene (P10) is hatched. The unrelated gene CDK5RAP1 is not filled. The black scale bars indicate either 50 kb or 10 kb.

In total therefore we have identified 12 distinct genes in the chicken genome. Details of these genes are provided in Table 1 and a detailed description of assignment of orthology is given below. As expected this list contains the 4 previously identified chicken genes, *CETP*, *PLTP*, *Ovocalyxin-36* and *TENP*. Six of the genes including *Ovocalyxin-36* and *TENP* are clustered on an approximately 60 kb locus on chromosome 20. Three further genes (including the orthologues of BPI and PLTP) are also found on chromosome 20. A further two genes, which we show below to encode orthologues of human *BPIL2*, (Mulero et al., 2002), are present on chromosome 1 while *CETP* is found on chromosome 11. The overall architecture of the PLUNC-like locus and its relationship to the human locus is shown in Fig. 1. This shows that 5 of the genes are transcribed in a centromeric-to-telomeric direction with the exception being *TENP*, which runs in the opposite direction. Analysis of this region of the zebra finch genome suggests the same orientation for the *TENP* gene in that species (results not shown) and this therefore represents the only reported instance of a member of this family within the conserved “PLUNC” locus running in the opposite direction with respect to all other genes. The general organisation of this region is very similar to a portion of the mammalian PLUNC locus encoding orthologous genes. *CDK5RAP1* is in the appropriate synteneic region of the chromosome and flanks the locus at the telomeric end. In the chicken locus *CDK5RAP1* is adjacent to *BPI.* In mammals these two genes are separated by approximately 5 Mb of genomic DNA and sequence syntenic to this portion of the mammalian genome is located approximately 8 Mb centromeric to this region in chicken suggesting a major reorganization has occurred in this portion of the chromosome.

### 3.2 Characterisation of gene structure

With systematic PCR-based sequencing of unknown genomic and cDNA segments within the predicted genes, the consensus transcript sequence, exon numbers and sizes were confirmed for most candidate chicken *BPI/LBP/PLUNC*-like genes. For some of these genes we were unable to predict the position and size of the presumptive 5′ and 3′ non-coding exons. The four gaps in the genomic assembly that were identified as potentially affecting exon predictions were bridged by PCR as described below.

Alignment of the complete sequence of chicken *BPI* with the genome assembly revealed that the 5′ end of the gene was missing. Amplification and cloning of this region by PCR allowed the identification of exons 3 and 4 and the confirmation of the size of exon 5.

The complete structure of the 5′ end of the *cBPILBP* gene was also absent from the assembly. To confirm this we aligned the sequence of the NCBI prediction (XP_417449.2) to the genome to generate locations of PCR primers. Amplification and cloning of this region by PCR allowed the identification of exons 2 and 3.

Alignment of the published cDNA sequence of *TENP* identified a gap in the assembly that included exons 9–12. PCR based cloning and sequencing confirmed the size and location of these exons.

Alignment of the cDNA of *LPLUNC6* (generated by fully sequencing EST CD216397) identified a gap at the 3′ end of the gene. Because there was a further EST (BM490026) with a different predicted exon organisation at the 3′ end (where exon 16 of >165 bp is replaced by 2 exons of 64 bp and >102 bp) we closed this gap to confirm that there were no other predicted exons in this region.

Table 1 summarises the gene information for the chicken *BPI/LBP/PLUNC*-like genes. The nomenclature that we have adopted in this paper was derived from the exon size information, physical location and phylogenetic analysis that is outlined in detail below. All candidate BPI/LBP/PLUNC-like protein genes have two LBP_BPI_CETP domains and most appear to have 16 exons. Importantly, there are no genes that encode SPLUNC proteins in the chicken genome. As was noted in our previous analysis of the gene family in mammals (Bingle et al., 2004) many of the exons have (near) identical sizes (Table 3). The two major exceptions to this rule in chicken are *CETP* and *P10* (the product of ENSGALG00000006693).

The cloning of chicken *CETP* has previously been described (Sato et al., 2007). Analysis of the *CETP* gene shows that the sequence that is equivalent to exon 5 in all other BPI/LBP/PLUNC-like genes is represented by 2 exons of 71 and 88 bases. This genomic organisation has previously been reported in mammalian *CETP* genes and our analysis shows that it is also found in the zebra finch (*Taeniopygia guttata*), the pipid frog (*Silurana tropicalis*) and zebrafish (*Danio rerio*) CETP orthologues (results not shown).

P10 shares some sequence homology with PLUNCs but lacks a complete predicted LBP_BPI_CETP domain. Sequencing the full-length of its supporting EST (BU136253) shows that it has a much shorter transcript length than other candidate PLUNCs. Genscan and BLASTp searches within the region identified an alternatively spliced prediction of 6 exons (XP_001235157), two of which 61 bp and 92 bp, are shared with the EST sequence. This prediction in many ways produces a more “PLUNC” like product but there is no EST support for this transcript and we were unable to amplify the corresponding transcript by PCR from pooled cDNAs (results not shown). As neither of the two gene predictions appear to encode a complete LBP_BPI_CETP domain it is likely that this gene represents an expressed pseudogene.

### 3.3 Comparative analysis

**Fig. 2:**
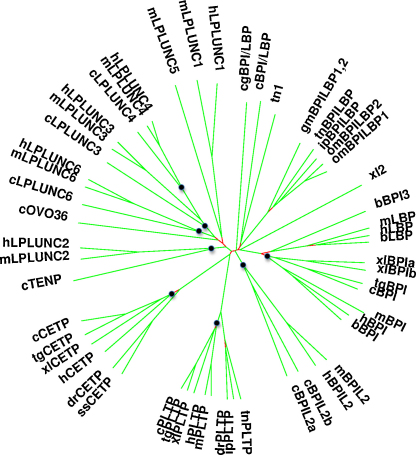
Unrooted phylogenetic tree of BPI/LBP/PLUNC-like family members. An unrooted phylogenetic tree of chicken BPI/LBP/PLUNC-like proteins was generated as described using additional two-domain containing proteins from a range of species. Where a chicken protein is identified as a 1:1(or a 2:1 for cBPIL2a/2b) orthologue of a human protein the position of the branchpoint marking the most recent ancestral protein of the pair is marked with a small filled circle. The closer the circle is to the centre of figure, the more dissimilar is the orthologous pair. The distances that must be traced along the tree to connect two proteins are related to the sequence similarity of the proteins. Example sequence identities to provide scale are hLPLUNC4: mLPLUNC4 at 86%, hLPLUNC4: cLPLUNC4 at 58%, cLPLUNC4: cLPLUNC3 at 40% and cLPLUNC4; cLBP/BPI at 20%. Branches with bootstrap values <95% are coloured red; the detailed branching order is uncertain in these regions. The following sequences were retrieved from public databases and used for the analysis. Zebra finch (*Taeniopygia guttata*). tgBPI, XP_002192404.1; tgCETP, XP_002197982.1; tgPLTP, XP_002191962.1. Zebra fish (*Danio rerio*). drPLTP, NP_001003519.1; drCETP, NP_001007361.1. Puffer fish (*Tetraodon nigroviridis*). tnBPILBP, CAF96904.1; tn1, CAG03233.1; tnPLTP, CAF90160.1. Atlantic cod (*Gadus morhua*). gmBPILBP1, AAM52335.1; gmBPILBP2, AAM52336.1. Trout (*Oncorhynchus mykiss*). omLBPBPI1, NP_001118057.1; omLBPBPI2, NP_001117670.1. Catfish (*Ictalurus punctatus*). ipBPILBP, AAX20011.1. Pacific oyster (*Crassostrea gigas*) cgLBP/BPI, AAN84552.1. Pipid frog (*Xenopus laevis*). xlCETP, NP_001088804.1; xlBPI1a, NP_001089628.1; xlBPI1b, NP_001086208.1; xlPLTP, NP_001087767.1; xl2, NP_001088621.1. Cow (*Bos taurus*). bBPI3, XP_592903.2; bLBP, NP_001033763.1; bBPI, NP_776320.1. Human (*Homo sapiens*). hLBP, NP_004130.2; hBPI, NP_001716.2; hCETP, NP_000069.2; hPLTP, AAH19847.1; hLPLUNC1, AAH08429.1; hLPLUNC4, NP_872325.2; hLPLUNC3, NP_872599.1; hLPLUNC2, NP_079503.1; hLPLUNC6, NP_777557.1; hBPIL2, NP_777592.1. Mouse (*Mus musculus*). mBPI, Q67E05.1; mLPLUNC1, NP_700467.2; mLPLUNC5, NP_659139.2; mLPLUNC3, NP_919338.2; mLPLUNC2, NP_079907.2; mLPLUNC6, Q8BU51.2; mBPIL2, NP_808440.2; mPLTP, AAH03782.1; mLPLUNC4, NP_001030047.2; mLBP, NP_032515.2. (For interpretation of the references to colour in this figure legend, the reader is referred to the web version of the article.)

Phylogenetic analysis of chicken BPI/LBP/PLUNC-like proteins with those of human, mouse, cow, zebra finch, *Xenopus laevis*, oyster and some fish species clearly identifies the presence of chicken orthologues of CETP, PLTP, BPI and BPIL2 (Fig. 2). The chicken genome encodes two *BPIL2* related genes which we have designated BPIL2a and BPIL2b (Fig. 2). The exon sizes of these two genes are almost identical and the proteins that they encode are 46% identical. The 5 complete genes present within the “PLUNC” locus are most closely related to each other in terms of sequence identity. Two of these genes are *Ovocalyxin-36* and *TENP*. Assignment of orthology to these 5 proteins is somewhat difficult due to the low sequence similarity that they share with the mammalian proteins. However, on the basis of phylogeny three of the proteins can be assigned as 1–1 orthologues of hLPLUNC3, hLPLUNC4 and hLPLUNC6. Our assignment of orthology is also supported both by exon size conservation and position within the locus. For example LPLUNC4 genes have a characteristically small exon 3 (63 bp) that is found in all species (Bingle et al., 2004). Consistent with our observation from other species the sequence similarity of hLPLUNC4 and cLPLUNC4 is strongest of all (Bingle et al., 2004). The orthology of TENP and Ovocalyxin-36 is less clear but on the same basis we would suggest that TENP is a divergent orthologue of LPLUNC2. The fact that Ovocalyxin-36 is located next to TENP suggest that they may have evolved by duplication from an ancestral LPLUNC2-related gene. TENP has the least conserved exon sizes of any of the chicken genes.

Finally there remains an outlier protein, which, despite a fairly conserved exon size pattern, does not appear to have any clear orthologous relationship to BPI, LBP or to any mammalian PLUNC proteins. On this basis we have given this protein the designation cBPI/LBP to show that it is related to but distinct from member of these sub-branches of the wider family.

One of the important outcomes from this phylogenetic analysis is that it fails to identify a chicken protein that is clearly orthologous to LBP. In all mammals the *LBP* is located within a few kb of the gene encoding BPI (Weiss, 2003; Bingle and Craven, 2004). *LBP* is also highly expressed in the liver and is represented by hundreds of mammalian ESTs. Genomic databases contain no additional protein predictions (either complete or partial) and we could identify no additional predicted related proteins when the chicken EST database was examined using tblastn. A clear LBP orthologue is also absent from the zebra finch genome (results not shown). Our conclusion is that there is no avian LBP orthologue. However, it should be pointed out that the region of the chicken genome that surrounds the presumptive location of this gene (immediately adjacent to BPI) has a number of gaps in the assembly and it remains a formal possibility that a chicken LBP gene is located in a region that has been incorrectly assembled. However no clear LBP orthologues exists in species that diverged prior to the avian/reptilian branch. Fish have only a single (or duplicated) BPI/LBP-like protein (Inagawa et al., 2002; Kono and Sakai, 2003), while *X. laevis* (and *S. tropicalis*, results not shown) has two distinct BPI/LBP-related proteins both of which group in the BPI branch of the tree (Fig. 2).

**Fig. 3:**
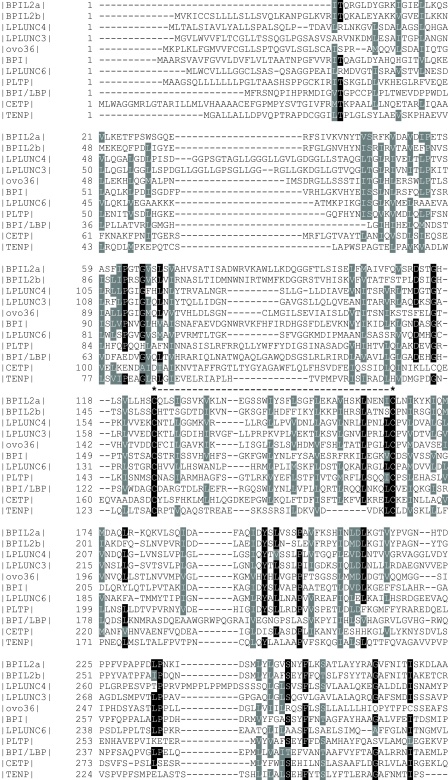

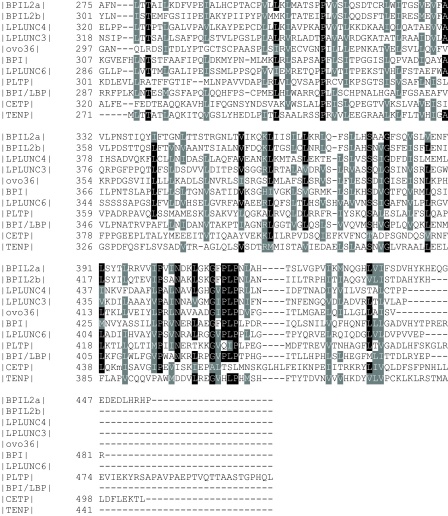
Multiple sequence analysis of chicken BPI/LBP/PLUNC-like proteins. The predicted amino acid sequences of chicken BPI/LBP/PLUNC proteins were aligned using CLUSTALX. In the alignment, identical residues (found in >7 sequences) are indicated by the white on black background, whereas similar residues (conserved in >7sequences) have the grey background. Spaces introduced for maximum alignments are indicated by a -. The positions of the conserved cysteine residues and the disulphide bond are indicated by *----*. The numbers indicate the amino acids.

Multiple sequence alignment of the chicken proteins shows an extremely low degree of sequence conservation (Fig. 3), an observation that is also shown by the divergence of the chicken orthologues from the mammalian proteins shown by the phylogenetic tree. With exception of the conserved proline residue at the C-terminus and the two cysteine residues in the N-terminal domain, which in BPI form a disulphide bond that maintains the three-dimensional structure of the protein (Beamer et al., 1997), there is no absolute conservation. This is in general agreement with the observed low sequence similarity between PLUNCs in mammals (Bingle et al., 2004; Wheeler et al., 2007). We have previously shown that LPLUNC proteins, with the exception of LPLUNC4/LPLUNC3, have less than 30% sequence identity between paralogues (Bingle et al., 2004).

### 3.4 Expression analysis

As some of the chicken BPI/LBP/PLUNC-like genes did not have any cDNA or EST support we generated primer pairs that could be used for RT-PCR for confirmation of their expression. We excluded analysis of CETP and PLTP, which have previously been studied in a range of tissues (Sato et al., 2007; Saarelaa et al., 2009). An initial screening using pooled cDNAs from healthy tissues followed by cloning and sequencing confirmed transcription of all ten genes.

**Fig. 4:**
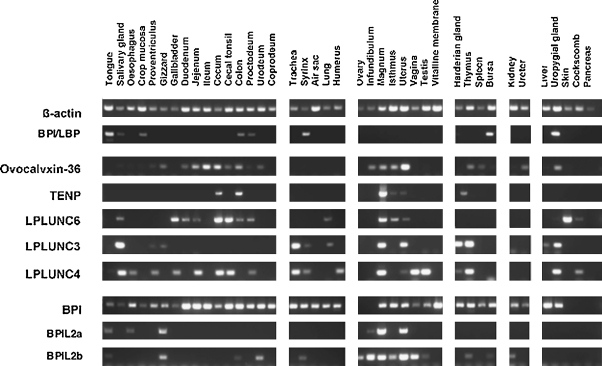
Expression analysis of chicken BPI/LBP/PLUNC-like proteins. Expression of chicken *BPI/LBP/PLUNC-*like genes was studied by PCR in multiple tissue samples using exon spanning primer pairs to each individual gene as described in the materials and methods section. Expression of β-actin (as a PCR control) was also monitored in the same samples.

Having established that we could amplify all of the chicken genes we then used a large cDNA panel to investigate the tissue specific expression of nine of the genes (not including the presumptive pseudogene, P10) (Fig. 4). It is clear that cBPI exhibits the widest distribution of expression with the other genes having a more restricted expression profile. The five proteins from the “LPLUNC” portion of the locus have a varied distribution pattern. Most strikingly all are expressed within tissues of the reproductive tract including tissues associated with egg laying. The other organ system that appear to expresses the majority of these genes is the gastrointestinal tract including the salivary gland. With the exception of cBPI, the respiratory tissues show a more restricted expression of the other PLUNC/BPI proteins. LPLUNC3 and LPLUNC6 are expressed in lung whereas LPLUNC3 and LPLUNC4 are expressed in the trachea. Interestingly the two BPIL2 genes exhibit a distinct expression pattern although both seem to be most highly expressed in tissues from the reproductive tract. In this analysis TENP appears to have the most restricted expression pattern. This initial transcriptional study serves two purposes. Firstly, it confirms that all of these genes do exist as transcribed RNA species and secondly, it shows that they exhibit distinct expression profiles suggesting that they are not co-regulated.

## 4 Discussion

Our systematic analysis confirms the existence of a portion of the PLUNC branch of the LBP_BPI_CETP domain containing superfamily in the chicken. Our previous analysis of wider PLUNC/BPI family had confirmed that TENP was a member but could not unequivocally place it on the PLUNC branch (Bingle et al., 2004). The subsequent identification and cloning of Ovocalyxin-36 had confirmed that additional related genes were present in the chicken genome (Gautron et al., 2007). Our analysis confirms that these two proteins are avian members of the “PLUNC” branch of the family and that an additional 3 related genes exist. The clustering of these seven structurally similar genes adjacent to *BPI* adds support to the hypothesis that PLUNCs arose from a series of duplication events (Bingle et al., 2004; Wheeler et al., 2007). Specifically, the absence of members of the *SPLUNC* branch of the family and the *LPLUNC1*/*LPLUNC5* arm of *LPLUNCs* in chicken implies that these proteins arose only after the speciation of mammals.

**Fig. 5:**
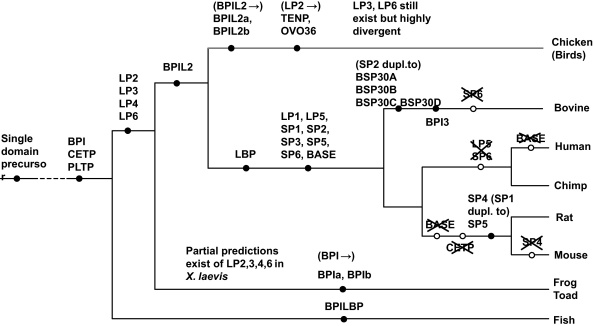
Schematic representation of the evolution of the BPI/LBP//PLUNC-like genes. The tree illustrates the evolution of the wider BPI/PLUNC-like gene family during vertebrate evolution. Fish represent the most divergent vertebrate ancestors in which clear orthologues exist. Black filled circles represent the earliest origin of clearly defined orthologues. Open circles with crossed out symbols represent gene loss in a lineage.

Our identification of the chicken PLUNC/BPI repertoire along with further analysis of additional genes from other species allows us to make some further suggestions as to the evolutionary origins of the wider gene family. This is represented in the schematic tree shown in Fig. 5. This schematic suggests that the family likely arose from a single domain containing precursor gene as both single and two-domain containing proteins are found in invertebrates (Kolodziejczyk et al., 2008; Gonzalez et al., 2007; Barker et al., 2008). Common ancestors of the vertebrates appear to have evolved PLTP, CETP and a BPI-like molecule as these proteins are found in a range of fish. In some fish, for example atlantic cod and trout, the BPI-like protein encoding gene has undergone duplication to yield two closely related molecules (Inagawa et al., 2002; Kono and Sakai, 2003). We suggest that LPLUNCs arose before the divergence of the synapsids (leading to mammal-like reptiles) and the sauropsids (leading to birds and reptiles) and are essentially common to all vertebrates. Pipid frogs (*S. tropicalis* and *X. laevis*) appear to have a number of genes (which are partially supported by EST sequences), corresponding to proteins with similarity to LPLUNC2, LPLUNC3, LPLUNC4 and LPLUNC6. We further propose that BPIL2 proteins arose after this divergence but before the therian divergence. The most significant expansion of the wider family appears to have arisen within the therian linage. Here we see the appearance of LBP as well as the significant expansion of the PLUNC sub-family of proteins including the evolution of LPLUNC1/5 and the SPLUNC branch. Both marsupials (grey short tailed opossum) and monotremes (platypus) have both groups of proteins. We have used RT-PCR with primers based on gene predictions for LPLUNC1, SPLUNC1 and SPLUNC2 to generate partial sequences for all three of these proteins from marsupials (results not shown) and a partial wallaby LPLUNC1 protein has been identified (Kwek et al., 2009). This schematic also highlights the diversification that has occurred in the SPLUNC portion of the family during the evolution of the eutherian mammals. We, and others, have previously highlighted this divergence, which includes examples of, expansion of paralogues, generation of pseudogenes and gene loss (Bingle and Craven, 2002; Bingle et al., 2004; Wheeler et al., 2007; Mulero et al., 2002). This divergence is also accompanied by loss of sequence conservation between orthologous proteins.

The absence of a presumptive LBP orthologue in chicken has important implications for the LBP-mediated portion of the LPS handling pathways. In mammals, LBP transfers LPS to CD14 on the surface of inflammatory cells, which in term transfers LPS to TLR-4 via MD-2 (Palsson-McDermott and O’Neill, 2004). Unlike most other TLRs, activation of TLR-4 initiate intracellular induction of two pathways with opposing effects: the MyD88/TIRAP pathway leads to activation of NF-κB and AP1 (Akira and Takeda, 2004); while the TRAM/TRAF pathway leads to IRF3-mediated inhibition of NF-κB release and production of IFNβ (Tanimura et al., 2008; Kagan et al., 2008). Although the chicken appears to have orthologues to MD-2, TLR4 (Malek et al., 2004) and CD14 (Keestra and van Putten, 2008; Wu et al., 2009), it seems to lack functional orthologues for TRAM, TRAF, TRIf and IRF3 (Keestra and van Putten, 2008; Cormican et al., 2009), supported by failure of LPS-induced INFβ production via the TRAM/TRAF pathway (Keestra and van Putten, 2008). The implication of this observation is that the MyD88/TIRAP signalling pathway has arisen later in evolution, and may explain the observations that birds and some fish species are more resistant to endotoxic shock than mammals (Adler and DaMassa, 1979; Berczi et al., 1966; Iliev et al., 2005).

From this study it is clear that chicken PLUNCs are expressed in a wide range of tissues, many of these are sites where mucosal/innate immunity is expected to play an important role. Our analysis represents expression at steady state and provides no indication of induction or regulation during infection and inflammation.

BPI, most abundantly expressed on neutrophils and eosinophils in human (Levy, 2000), is expressed almost constitutively in the chicken tissues, as confirmed by consistent results with different primer sets and multiple negative controls. Although tissue contamination by granulocytes is a possibility, the intensity of amplification observed is higher than would be expected as the transcriptional activities of granulocytes are highest in the bone marrow and quite low in the peripheral circulation. It can therefore be concluded that BPI is expressed in tissue types different from the major sites of expression in human. Recently, additional expression sites of BPI have been noted including inducible expression in gut, respiratory and reproductive epithelium (Canny and Levy, 2008; Canny et al., 2002, 2006). A similar interspecies difference in expression has also been observed with murine BPI, which is strongly expressed in the testis, in addition to bone marrow granulocytes (Lennartsson et al., 2005).

As suggested in the introduction there is little known of the expression of the other chicken PLUNC proteins. Ovocalyxin-36 was found to be expressed in the infundibulum, magnum, isthmus, and uterus of the female reproductive tract (Gautron et al., 2007), although these studies did not analyse expression in a comprehensive set of tissues. It has also been reported in proteomic studies of chicken eggs (Mann, 2008). More recent studies have also confirmed that members of this gene family are found in tissues associated with egg production and within eggs themselves (D’Alessandro et al., 2010; Dunn et al., 2009; Jonchère et al., 2010). Our studies suggest that rather than being expressed exclusively in reproductive tissues associated with egg production, the protein is abundantly expressed in the digestive tract. The other previously studied PLUNC protein, TENP, was initially found to be expressed in the adult and embryonic brain and retina, although again this study did not include a wide range of tissues (Yan and Wang, 1998). Consistent with our expression data, TENP protein has also been shown to be present in the magnum, the part of the female reproductive tract responsible for secretion of egg white as well as in egg white (Mann, 2008). Although genes in the “LPLUNC” portion of the family exhibit a widespread distribution a number of the other genes, principally LPLUNC4 and LPLUNC6 are also expressed in the digestive tract. Currently there is no published evidence that mammalian orthologues of these genes are expressed in these tissues.

As the original murine PLUNC was found to be restricted to the upper respiratory tract and oral cavity (Weston et al., 1999) and we have subsequently shown that other family members are often highly expressed in this region (Bingle and Craven, 2002; Bingle et al., 2005, 2009, 2010), we were interested in identifying chicken PLUNCs that are expressed in these tissues. Our analysis suggests that a number of these genes are expressed in these tissues but that none of them exhibit specificity for the respiratory tract. Again, this may not be totally unexpected. The two most highly expressed pulmonary-restricted PLUNCs in human and mouse, are SPLUNC1 and LPLUNC1 (Bingle et al., 2005, 2010; Barnes et al., 2008), which are both absent in chicken. It remains an open question as to why the SPLUNC and LPLUNC1/5 arms of the wider protein family is absent below the therian lineage. It is tempting to speculate that these genes have co-evolved with the development of the alveolarised lung that first developed in the monotreme lineage (Ferner et al., 2009). Birds have previously been noted to lack other additional pulmonary genes. For example genes for *Surfactant protein B* and *Surfactant protein C*, both critical for the function and defence of the alveolarised lung have not as yet been reported in the chicken genome (Hogenkamp et al., 2006; Hughes, 2007). Recent studies have suggested that SPLUNCs may function as surfactant molecules (McDonald et al., 2009; Gakhar et al., 2010), and perhaps these functions are not essential for the avian lifestyle.

## 5 Conclusions

In summary, our comprehensive bioinformatic and expression analysis has identified a total of five PLUNC homologues in chicken, all of which appear to be LPLUNCs. They are structural orthologues of LPLUNC2, 3, 4 and 6. One additional avian-specific LPLUNC (BPI/LBP) has also been identified. Chicken orthologues of BPI, CETP, PLTP, BPIL2 have also been confirmed. In contrast to mammals, there appears to be no LBP in avians, which possibly attributes to the higher LPS sensitisation threshold in birds relative to mammals. Additional expression and functional analysis of this complex gene family are required to elucidate their putative host defence function.

## Figures and Tables

**Table 1:** Chicken genes and predicted proteins containing LBP_BPI_CETP domains.

Description	Peptide ID	Ensembl gene ID	Location	Sequenced ESTs	Gap closure required
BPIL2a	XP_425484	ENSGALG00000012602	Chr1:49,966,319-49,998,072	DT655663	–
BPIL2b	XP_001234743	ENSGALG00000023111	Chr1:55,310,190-55,322,401	BU432695	–
CETP	NP_001029986	ENSGALG00000001234	Chr11:611,151-616,777	–	–
BPI/LBP	XP_417449.2	ENSGALG00000021025	Chr20:9,964,037-9,966,515	–	Yes
Ovocalyxin-36	NP_001026032	ENSGALG00000006662	Chr20:10,238,414-10,246,457	CD217547	–
TENP	NP_990357	ENSGALG00000006666	Chr20:10,247,698-10,251,922	DT657764	Yes
LPLUNC6	XP_417463.2	ENSGALG00000006674	Chr20:10,253,409-10,260,128	CD216397	Yes
LPLUNC3	XP_425718	ENSGALG00000020982	Chr20:10,263,408-10,268,038	BU362274	–
LPLUNC4	–	ENSGALG00000006679	Chr20:10,270,264-10,275,479	–	–
Protein 10	–	ENSGALG00000006693	Chr20:10,279,239-10,282,757	BU136253	–
BPI	XP_417465.2	ENSGALG00000006756	Chr20:10,293,925-10,301,731	BM491427	Yes
PLTP	XP_425722	ENSGALG00000006894	Chr20:10,491,966-10,496,062	–	–

**Table 2:** Oligonucleotide primers for each chicken gene.

Gene	Name	Sequence (5′–3′)	Use
BPI	RT-F	GAGTGGTGCTGGATGTGTTCCTGGT	RT-PCR
	RT-R	ACGGCGCTATAGAAGAGGTTG	RT-PCR
	GF-1	GAGTGGTGCTGGATGTGTTTC	Genomic PCR
	GF-2	GGGGAAGGTGCACTATGAGAT	Genomic PCR
	GR-1	ACGGCGCTATAGAACAGGTTG	Genomic PCR
	SeqF	CCCACCCCACTGTACTGTCT	Sequencing
	SeqR	CATGGGAGAACCTGTGTCCT	Sequencing
Ovocalyxin-36	RT-F	ACGTCTCAATGCTGTTGCAG	RT-PCR
	RT-R	ATCCTGACCGGATCTCCTCT	RT-PCR
TENP/LPLUNC2	RT-F	TCCGAGCACTTCTACACCAG	RT-PCR
	RT-R	CCAAGATCAGGGTTGGAAGA	RT-PCR
	GF-1	AACTGCCAGCTCCAGTACC	Genomic PCR
	GR-1	TCCGAGCACTTCTACACCAG	Genomic PCR
BPIL-2R1	RT-F	ACACAGACCCTCCCTTTGTG	RT-PCR
	RT-R	TGTTGTCAGATTCCCCGTAA	RT-PCR
BPIL-2R2	RT-F	TTCCGACCGTACATTGACAT	RT-PCR
	RT-R	GTGGCCGTTAGCTTCATCAT	RT-PCR
LPLUNC6	RT-F	GTGCTGAATGAGAGCGATGT	RT-PCR
	RT-R	ATTGAAAAGCCAAGGGGAGA	RT-PCR
LPLUNC4	RT-F	GCTGCTGAAAATCGCAGTTC	RT-PCR
	RT-R	CAAACTGAGCCAGCAAGGAC	RT-PCR
LPLUNC3	RT-F	GTTCTGGACATCACCCCATC	RT-PCR
	RT-R	TACATCAGCCAAGTCCACCT	RT-PCR
Protein 12	RT-F	GTGTGTCATGGAAGGTGCTG	RT-PCR
	RT-R	CTTTTTTATTGCCAGGAGCA	RT-PCR
Protein 11	RT-F	CACTCACATGGGAAGTGGAC	RT-PCR
	RT-R	ACCGCCAGGTCCCTGATAC	RT-PCR
	GF-1	ATGGACATTGGCGTCACAG	Genomic PCR
	GR-1	CAGGTGGCATTGAGCTGGAT	Genomic PCR
	SeqF	ATCCTTCTGCTCCTCAGTTG	Sequencing
	SeqR	ACAGCCACATCCCCCGGGTT	Sequencing
β-Actin	RT-F	ACCCTGTCCTGCTTACTGAGG	RT-PCR
	RT-R	TCCCAATGGTGATCACCTGCC	RT-PCR

**Table 3:** Exon sizes of chicken BPI/LBP/PLUNC-like genes. *Bold* exon sizes were determined after aligning cDNA sequences to sequenced gaps present in the chicken genome assembly.

Exon no.	1	2	3	4	5	6	7	8	9	10	11	12	13	14	15	16
BPIL2a		15	112	129	156	64	61	92	174	54	171	68	43	64	77	>368
BPIL2b	>55	127	112	129	156	64	61	89	174	54	171	68	43	64	77	>123
CETP		170	115	135	71 + 88	70	61	92	180	51	168	68	43	70	86	>533
BPI/LBP	>149	***180***	***115***	129	156	64	61	92	177	60	168	68	43	64	77	
Ovocalyxin-36		239	115	105	156	64	64	92	174	54	168	68	43	64	77	>481
TENP	>30	117	100	105	135	46	61	92	***177***	***42***	***165***	***68***	46	64	77	>279
LPLUNC6		147	100	105	147	64	61	92	177	54	171	68	46	***64***	***77***	>165 (64/>112)
LPLUNC3	>118	114	52	105	141	64	61	92	183	54	171	68	43	64	77	
LPLUNC4	>112	63	100	105	144	61	61	92	213	54	171	68	43	64	77	
P10		189		329			61	92	43	124						
BPI		116	***115***	***129***	***156***	64	64	92	177	60	168	68	43	64	77	>545
PLTP	>46	119	100	129	156	64	64	92	177	60	165	68	43	64	77	>300
